# The knock-on effects of COVID-19 pandemic on the supply and availability of generic medicines in Ethiopia: mixed methods study

**DOI:** 10.1186/s12913-023-09535-z

**Published:** 2023-05-20

**Authors:** Zeleke Mekonnen, Tsegaye Melaku, Gudina Terefe Tucho, Mohammed Mecha, Christine Årdal, Marianne Jahre

**Affiliations:** 1 Institute of Health, Jimma University, Jimma, Oromia Ethiopia; 2 Antibiotic Resistance and Infection Prevention, Norwegian Institute of Public Health, Oslo, Norway; 3 Department of Accounting and Operations Management, BI Norwegian Business School, Oslo, Norway

**Keywords:** Availability, Chronic disease medicines, COVID-19, Essential medicines, Inventory management, Paracetamol products, Stock status

## Abstract

**Background:**

COVID-19 pandemic posed a major impact on the availability and affordability of essential medicines. This study aimed to assess the knock-on effects of the COVID-19 pandemic on the supply availability of non-communicable chronic disease (NCD) medicines and paracetamol products in Ethiopia.

**Methods:**

A mixed methods study was conducted to assess the supply and availability of twenty-four NCD drugs and four paracetamol products listed on the national essential medicines list for hospitals. Data were collected from twenty-six hospitals located in seven zones of Oromia region in the southwestern part of Ethiopia. We extracted data on drug availability, cost and stock out for these drugs between May 2019 and December 2020. The quantitative data were entered into Microsoft Excel and exported to statistical package software for social science (SPSS) version 22 (IBM Corporation, Armonk, NY, USA) software for analysis.

**Results:**

The overall mean availability of selected basket medicines was 63.4% (range 16.7% to 80.3%) during the pre-COVID-19 time. It was 46.3% (range 2.8% to 88.7) during the pandemic. There was a relative increase in the availability of two paracetamol products [paracetamol 500 mg tablet (67.5% versus 88.7%) and suppository (74.5% versus 88%)] during the pandemic. The average monthly orders fill rates for the selected products range from 43 to 85%. Pre-COVID-19, the average order fill rate was greater or equal to 70%. However, immediately after the COVID-19 case notification, the percentage of order(s) filled correctly in items and quantities began decreasing. Political instability, shortage of trained human resources, currency inflation, and limited drug financing were considered as the major challenges to medicine supply.

**Conclusion:**

The overall stock out situation in the study area has worsened during COVID-19 compared to pre-COVID-19 time. None of the surveyed chronic disease basket medicines met the ideal availability benchmark of 80% in health facilities. However, availability of paracetamol 500 mg tablet surprisingly improved during the pandemic. A range of policy frameworks and options targeting inevitable outbreaks should exist to enable governments to ensure that medicines for chronic diseases are consistently available and affordable.

## Background

The coronavirus disease 2019 (COVID-19) pandemic constitutes the biggest global public health threat in a century, with overwhelming health and socioeconomic challenges [1]. Since it was declared a world pandemic by the World Health Organization (WHO) in March 2020, the virus has continued to spread to different parts of the world. It has created a vicious cycle affecting all facets of life, including political, economic, social, or technological security [2]. The situation is extremely difficult in humanitarian, fragile, and low-income countries, where health and social systems are already weak and resources are deficient [3]. The United Nations Secretary-General, António Guterres explained the magnitude of the current crisis as:

*“This is not a financial crisis. This is a human crisis. This is not a question of just bringing liquidity to the financial systems, which, of course, is necessary. We need to support directly those that lose their jobs, those that lose their salaries, the small companies that cannot operate anymore, all those that are the fabrics of our societies. We need to make sure that we keep thousands afloat, we keep small companies afloat, and we keep all societies afloat.”*

The health industry, which is the epicenter of virus control, has been the worst affected. As the pandemic continues to spread across the globe, it has exposed supply chains and logistics vulnerabilities. It has affected the supply chain's key elements (i.e., upstream, internal, and downstream components), including the information systems that interlink them [4–6].

Multi-faceted impacts were observed in the health system of low and middle-income countries (LMICs) like Ethiopia. The pharmaceutical systems in Ethiopia were grossly affected by the pandemic, limiting access to essential medicines at public and private health facilities [7–9]. Its knock-on effect on the availability of essential medicines is intensified, highlighting the fragility of supply and logistics management [8, 10]. These supply disruptions or medicine shortages could occur due to temporary lockdowns of manufacturing sites, travel restrictions impacting exports, export bans, increased demand, stockpiling by hospitals or by individual citizens, or at the country level [11–13].

These disruptive effects of COVID-19 have put a tremendous strain on the worldwide supply of medical products, increasing the risk of shortages [14, 15]. This applies to classes of medicines for which unavailability or interruption to treatment could cause significant health impacts or are expected to be needed in greater quantity during this pandemic such as HIV medicines, cardiovascular disease (CVD) drugs, and antidiabetics, vaccines, and contraceptives [15–22].

In addition to the weak supply of medicines, reallocation of resources to COVID-19 could interrupt the continuum of chronic disease care [23]. A survey conducted by WHO showed that clinical care for diabetes mellitus, chronic obstructive pulmonary disease, hypertension, heart disease, asthma, cancer, and depression were highly affected [16, 24]. One of the main reasons for discontinuing services was a shortage of medicines, diagnostics, and other technologies [25–28]. This situation is of significant concern because people living with NCDs are at higher risk of severe COVID-19-related illness and death [24].

To this end, understanding the effect of the pandemic on the availability of essential medicines has paramount importance for efficiently managing supply chain disruptions in the context of the inevitable pandemic in the future. Ensuring access to health services is the cornerstone of successful health response. Therefore, this study aimed to assess the knock-on effect of COVID-19 on the availability and stock status of antidiabetics, CVD medicines, and paracetamol products in Ethiopia. It also identified the challenges, lived experience, and possible interventions to prevent supply interruptions at service delivery points in the study area.

## Methods

### Study design and setting

**Fig. 1:**
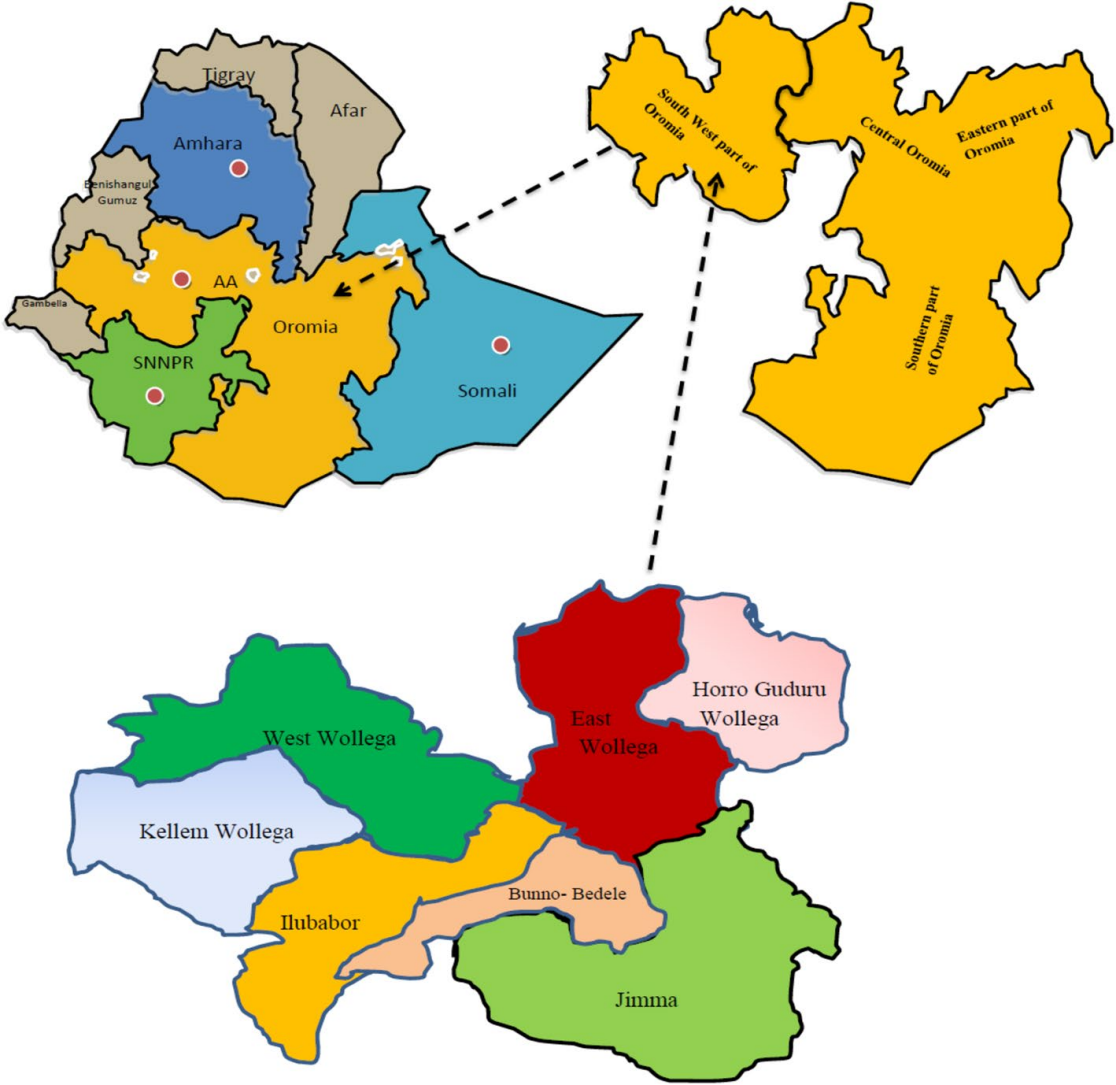
Map of Ethiopia and study area (designed using Canva design; https://www.canva.com/design/DAFddjWPsAs/JOlltFCxb-GrvTh0y_Qt3w/edit)

A mixed methods study was conducted to assess the supply and availability of twenty-four NCD drugs and four paracetamol products listed on the national essential medicines list for hospitals. The health facilities (hospitals) and public supply agencies involved in medicine supply found in seven (7) zonal administrations in Ethiopia were included in the study (Fig. 1). The study area was one of those affected by COVID-19 cases, as well as far from the capital, which could have an impact on supply and logistics during the outbreak. The study adopted the qualitative case study design, which is used as an empirical inquiry that investigates a contemporary phenomenon in depth and within its real-life context to know how COVID-19 affected the availability and stock status of selected basket medicines. The study included data from May 01, 2019 to December 31, 2020.

### Study population and sample size

Based on the Logistic Indicators Assessment Tool (LIAT) [29] for the assessment of the availability of pharmaceutical commodities, it is recommended to include 15% of the health facilities in the identified study area. However, in this study, all the hospitals in the seven zonal administrations and two government-owned branch pharmaceutical supply agencies found in the southwestern part of Ethiopia were included. There are a total of 31 different level hospitals found in these zonal administrations. Of these hospitals, two of them are privately-owned health institutions and usually were not supplied by the government. Depending on the level of facilities, each hospital follows specific standard treatment guidelines developed by the Ministry of Health for the management of disease conditions such as diabetes and cardiovascular disease. The average estimated number of patients on chronic disease follow-up ranged from 500 in primary care to 3500 in tertiary care per year. All the hospitals provide basic inpatient and ambulatory services. Hospitals are expected to provide various types of clinical services depending on their level and catchment population. The For qualitative data health workers involved in the drug supply and logistics management of health commodities in selected facilities were included. These were store managers, pharmacy service directors, and medicine forecasting officers and, supply agency managers.

### Data collection process

The quantitative and qualitative data were collected using three separate tools: the Logistics System Assessment Tool (LSAT) [30], Inventory Management Assessment Tool (IMAT), and the LIAT [29]. The data collection involved collecting primary data from professionals in hospitals and supply agencies. A comprehensive document review on supply chain processes improvement was also conducted. Preceding the key informant interviews on the supply chain processes and the availability of those medicines in the hospitals, documents were reviewed and explored the magnitude of the problem related to the stock status and inventory management practices for antidiabetic, CVD medicines, and paracetamol products.

The key informant interviews were conducted; using questions adapted from the LSAT [30], with logistics and pharmacy professionals to understand the challenges facing the logistics systems and obtain a description of the stock status of selected chronic disease medicines and paracetamol products before and during COVID-19 pandemic. Those medicines were paracetamol products, antidiabetics, CVD medicines listed on the national essential drug list. These pharmaceutical products were selected for the evaluation based on the following reasons. (1) Patients living with chronic diseases such DM and CVD are high risk patients to contract COVID-19 and thus lack of access to the medications will affect the patient management. (2) Paracetamol products are considered as one of the vital medications to manage patient with COVID-19. The increase in number of COVID-19 cases expected to affect its availability at the health facility due to increased use. The data collectors interviewed persons responsible for managing the supply of these medicines at each service delivery point using a semi-structured questionnaire and physical inventory of those medicines (to verify the availability or non-availability of items at both service delivery points (SDPs), stores, and supply agencies warehouse) was conducted. The two-day training was given to the data collectors regarding the study's objectives and how to collect the data. During the data collection process, there was close supervision by the investigators to ensure consistency and completeness of the data.

### Data processing and analysis

The data from primary and secondary sources were aggregated together. It was analyzed using both qualitative and quantitative techniques. The quantitative data were entered into Microsoft Excel and exported to statistical package software for social science (SPSS) version 22 (IBM Corporation, Armonk, NY, USA) software for analysis. The data was analyzed using descriptive and inferential statistics at statistical significance of p < 0.05. Analysis of qualitative data involved rigorous reading of transcripts to identify key themes, and data were analyzed using a thematic approach. Audio-recorded interviews were transcribed and the raw data was categorized under coded themes and sub-themes. Two of the authors coded the data (TSM and ZM). The average duration of the interviews was 15 min. It was transcribed and summarized manually and presented in the form of narration.

### The outcome measure, and indicators

In this study, the stock status and inventory management practices of antidiabetics, CVD medicines, and paracetamol products during the COVID-19 pandemic were measured by using a selected supply chain management performance measuring matrix by function. This utilizes the selected indicators for each logistics activity. This assessment revealed the sum of stock levels available at the peripheral warehouse (two Ethiopian Pharmaceutical supply agency hubs), and service delivery points (hospitals). Data from Logistics Management Information System (LMIS) tools for the last eight months before the COVID-19 outbreak was compared with its one year status during COVID-19. This period was selected purposively. In Ethiopia, the month of May/June is when the government wraps up fiscal year activities and prepares for the New Year budget. As a result, we only have eight months counting back from December 31^st^, 2019 (COVID-19 outbreak) to know the most recent information about the stock.

#### Availability of selected products

The percentage of selected basket medicines in the stock measures the system's effectiveness in maintaining a range of these products in the health facility at the time of the assessment.

It was calculated as:

$$\frac{\mathrm{The}\;\mathrm{total}\;\mathrm{products}\;\mathrm{in}\;\mathrm{stock}}{\mathrm{Total}\;\mathrm{number}\;\mathrm{of}\;\mathrm{products}\;\mathrm{in}\;\mathrm{the}\;\mathrm{study}}\;\times\;100$$

Product availability was assessed based on the WHO’s availability index. A product is available if available in the health facility providing a service on the day of the visit or during the specified period. The following ranges were used for describing availability: < 30%- very low; 30–49%—low; 50–80%—fairly high; > 80%—high [31, 32]. The assessment of availability took into account the level of health facility as well as the inclusion of the product on a specific facility's essential medicine list or procurement list. As a result, products that are not managed by the facility and are not available during the assessment were not considered in the availability assessment.

#### Order fill rate

In addition, order fill rate for the product was assessed. This indicator was used to measure the percentage of the difference between the amount ordered and the amount received for each pharmaceutical product in each month. This was calculated as:

$$1-\;\frac{\left[\mathrm{Quantity}\;\mathrm{ordered}\;-\;\mathrm{quantity}\;\mathrm{supplied}\right]}{\mathrm{Quantity}\;\mathrm{ordered}}\;\times\;100$$

#### Stock out the status

The stock status of selected CVD medicines, antidiabetics, and paracetamol products was assessed based on the monthly records of the day out of stock and order fill rates. The availability of selected drugs on the national EML was measured by quantifying the number and duration of stock out reported by each hospital. The twenty-month aggregate data was split into two groups, pre-COVID-19 and during COVID-19 for each class of product.

#### Unit cost changes

The average unit cost of medicine was evaluated at service delivery points. Accordingly, the range of changes was compared between pre-COVID-19 time and during the COVID-19 pandemic.

## Results

### Quantitative data

#### General information about the study setting

From a total of the thirty-one hospitals found in the southwestern part of Oromia regional state in Ethiopia, twenty-six (*n* = 26) hospitals and two (*n* = 2) Ethiopian pharmaceutical supply agency branches were included in this study. Two hospitals found in the west Wollega zone were excluded, as they are not government-owned service delivery points. The two hospitals found in Horro Guduru Wollega were excluded due to the political situations and security issues in the area. The last excluded hospital was newly opened. Among the included hospitals, two (2) were tertiary/specialized teaching hospitals, fifteen (15) general hospitals and nine (9) were primary hospitals. Tertiary teaching/specialized hospitals and most general hospitals had two pharmacy professionals (pharmacist and/or druggist) at the medical store (, while primary hospitals had one pharmacy professional (pharmacist or druggist) at the medical store. One health facility medical store, which is a primary hospital, was managed by a nurse. The entire tertiary-teaching and general hospital medical store were managed by a pharmacist. Five of the nine primary hospitals stores were managed by the druggist. Most, 11(42.3) store managers had years of professional experience between 3 and 5 years. The medical store is a dedicated room with office where pharmaceutical products are stored before being dispatched to hospital pharmacies and dispensing for consumers/clients in the same health institution. The current stock status assessment included twenty-eight (28) essential medicines in different dosage forms. Of these, nineteen (19) of them were CVD drugs, six (5) of them were antidiabetics and the rest were paracetamol products (Table 1).

**Table 1:** Background information on the study sites, professionals, and drug products

Variables	Category	Frequency	Percentage
Facility type (*n* = 26)	Tertiary/teaching hospital	2	7.7
	General hospital	15	57.7
	Primary hospital	9	34.6
	Public supply agency (hubs)	2	-
Store manager (*n* = 26)	Pharmacist^b^	20	77
	Druggist^b^	5	19.2
	Nurse	1	3.8
Work experience of store manager (years) (*n* = 26)	≤ 2	6	23.1
	3–5	11	42.3
	> 5	9	34.6
Product categories (*n* = 28 drug formulations on EML)	CVD medicines	19	67.8
	Antidiabetics	5	17.9
	Paracetamol products	4	14.3
Antidiabetics (*n* = 5)	Insulin products	3	60
	Oral hypoglycemic agents	2	40
CVD medicines (*n* = 19)	ACEIs/ARB	3	15.8
	Beta- blockers	3	15.8
	Diuretics	4	21.1
	Statins	3	15.8
	Digoxin products	2	10.5
	Calcium channel products	2	10.5
	Others^a^	2	10.5

*ACEIs/ARB* Angiotensin Converting Enzyme Inhibitors/ Angiotensin Receptor Blockers, *CVD* Cardiovascular disease

^a^ *Methyldopa, Aspirin (81 mg); EML: Essential Medicine List*

^b^Pharmacists have a bachelor's degree in pharmacy and have received five years of training. Druggists, on the other hand, are pharmacy technicians who have received two years of training and typically support pharmacist services in hospitals

#### Distribution of selected basket medicines

The distribution of selected basket medicines among the three levels of the hospital was 98.2% in the two tertiary specialized hospitals, 92.6% in the 15 general hospitals, and 82.1% in the 9 primary hospitals. Overall, 89.1% of drugs from the three classes of medicines were included in the health facility's drug list. All of the paracetamol formulations were included in the procurement or drug list of all selected health facilities. Digoxin 0.25 mg/ml (injection) was included in the two (2) tertiary hospitals and some (26.7%) of the general hospital drug list. Losartan 20 mg and digoxin (0.25 mg/ml) injection were not managed by primary hospitals (Table 2).

**Table 2:** Distribution and inclusion of selected medicines to the facility’s drug list and/or procurement list

List of drug products	Tertiary/teaching hospital (*n* = 2) n (%)	General hospital (*n* = 15) n (%)	Primary hospital (*n* = 9) n (%)	Total (*n* = 26) n (%)
1. Insulin regular (soluble) 100units/ml	2	15(100)	9(100)	26(100)
2. NPH (insulin suspension) 100units/ml	2	15(100)	9(100)	26(100)
3. Insulin Isophane (biphasic) (30/70) 100units/ml	2	15(100)	9(100)	26(100)
4. Metformin 500 mg tablet	2	15(100)	9(100)	26(100)
5. Glibenclamide 5 mg tablet	2	15(100)	9(100)	26(100)
6. Aspiring 81 mg tablet	2	15(100)	9(100)	26(100)
7. Enalapril 5 mg tablet	2	15(100)	9(100)	26(100)
8. Captopril 25 mg tablet	2	15(100)	9(100)	26(100)
9. Losartan 20 mg tablet	1	2(13.3)	0	3(11.5)
10. Atenolol 50 mg tablet	2	15(100)	9(100)	26(100)
11. Propranolol 40 mg tablet	2	15(100)	5(55.6)	22(84.6)
12. Metoprolol 25 mg tablet	2	15(100)	2(22.2)	19(73.1)
13. Frusemide 40 mg tablet	2	15(100)	9(100)	26(100)
14. Frusemide 10 mg/ml in 2 ml(injection)	2	15(100)	7(77.8)	24(92.3)
15. Hydrochlorothiazide 25 mg tablet	2	15(100)	9(100)	26(100)
16. Spironolactone 25 mg tablet	2	15(100)	9(100)	26(100)
17. Atorvastatin 20 mg tablet	2	15(100)	9(100)	26(100)
18. Lovastatin 20 mg tablet	2	11(73.3)	3(33.3)	16(61.5)
19. Simvastatin 40 mg tablet	2	14(93.3)	4(44.4)	20(76.9)
20. Methyldopa 250 mg tablet	2	15(100)	9(100)	26(100)
21. Amlodipine 5 mg tablet	2	15(100)	7(77.8)	24(92.3)
22. Nifedipine 20 mg tablet	2	13(86.7)	8(88.9)	23(88.5)
23. Digoxin 0.25 mg tablet	2	15(100)	9(100)	26(100)
24. Digoxin 0.25 mg/ml in 2 ml(injection)	2	4(26.7)	0	6(23.1)
25. Paracetamol 500 mg tablet	2	15(100)	9(100)	26(100)
26. Paracetamol 100 mg tablet	2	15(100)	9(100)	26(100)
27. Paracetamol 250 mg/5 ml syrup	2	15(100)	9(100)	26(100)
28. Paracetamol 125 mg suppository	2	15(100)	9(100)	26(100)
Total^a^	55(98.2)	389(92.6)	207(82.1)	649(89.1)

^a^ *Denominator* = *number of facilities x total*

#### Stock status of selected basket medicines

##### Product availability

The stock status of CVD medicines, antidiabetics, and paracetamol products at each hospital was measured; this included a review of stock availability for both the stock levels during COVID-19 (January—December 2020) and stock levels for the eight months before COVID-19 (May—December 2019). The finding of the current study revealed that the overall availability of selected basket medicines was 63.4% (range 16.7% to 80.3%, standard deviation = 14.6) during the pre-COVID-19 time. It was 46.3% (range 9.7% to 88.7%, standard deviation = 19.8) during COVID-19. The outbreak created significant changes in the availability of CVD medicines and antidiabetics in the hospitals.

The availability of CVD medicines was high for Atenolol which was 80.3% before the COVID-19 outbreak and decreased to 58.7% during the pandemic. Methyldopa was fairly available before COVID-19 (56.3%) and its availability was decreased to 25.7% during the outbreak. The availability of diuretics was not significantly changed during COVID-19 as compared to their pre-COVID-19 availability data. Hydrochlorothiazide had equivalent availability (75.5% versus 75.7%) pre and during the COVID-19 time, respectively. The overall availability of insulin products showed significant differences before and during the COVID-19 pandemic. For example; Insulin Isophane (*Biphasic 30/70*) was fairly available before the pandemic. However, its availability at the hospitals was very low during COVID-19. Concerning the oral hypoglycemic agents, the availability of metformin was decreased nearly by half (64.4% versus 35.3%). Glibenclamide was fairly available at hospitals. Overall availability of digoxin injection and losartan was very low at hospitals.

The availability of paracetamol products was not significantly affected by the outbreak. Their availability lay between high to fairly high according to the WHO availability index. Yet, the availability of paracetamol 500 mg tablet (67.5% versus 88.7%) and suppository (74.5% versus 88%) showed an increased availability during the COVID-19 (Table 3).

**Table 3:** Availability of selected basket medicines in the public hospitals pre-COVID-19 and during COVID-19

**List of drug products**	Average availability			
	Before COVID-19 (%)	WHO Availability Index	During COVID-19 (%)	WHO Availability Index
1. Insulin regular (soluble) 100 units/ml	76.9	Fairly high	46.5	Low
2. NPH (insulin suspension) 100 units/ml	75.5	Fairly high	48.7	Low
3. Insulin Isophane (biphasic) (30/70) 100 units/ml	74.5	Fairly high	28.5	Very low
4. Metformin 500 mg tablet	64.4	Fairly high	35.3	Low
5. Glibenclamide 5 mg tablet	73.1	Fairly high	54.5	Fairly high
6. Aspiring 81 mg tablet	70.2	Fairly high	25.3	Low
7. Enalapril 5 mg tablet	67.3	Fairly high	41.7	Low
8. Captopril 25 mg tablet	70.8	Fairly high	55.5	Fairly high
9. Losartan 20 mg tablet	16.7	Very low	2.8	Very low
10. Atenolol 50 mg tablet	80.3	High	58.7	Fairly high
11. Propranolol 40 mg tablet	52	Fairly high	34.9	Low
12. Metoprolol 25 mg tablet	63.8	Fairly high	38.2	Low
13. Frusemide 40 mg tablet	78.9	Fairly high	49.1	Low
14. Frusemide 10 mg/ml in 2 ml(injection)	77.1	Fairly high	61.8	Fairly high
15. Hydrochlorothiazide 25 mg tablet	75.5	Fairly high	75.7	Fairly high
16. Spironolactone 25 mg tablet	71.2	Fairly high	67	Fairly high
17. Atorvastatin 20 mg tablet	55.3	Fairly high	43.9	Low
18. Lovastatin 20 mg tablet	59.4	Fairly high	41.2	Low
19. Simvastatin 40 mg tablet	60.6	Fairly high	42.1	Low
20. Methyldopa 250 mg tablet	56.3	Fairly high	25.7	Very low
21. Amlodipine 5 mg tablet	55.2	Fairly high	45.5	Low
22. Nifedipine 20 mg tablet	52	Fairly high	34.9	Low
23. Digoxin 0.25 mg tablet	54.3	Fairly high	39.1	Low
24. Digoxin 0.25 mg/ml in 2 ml(injection)	27.1	Very low	9.7	Very low
25. Paracetamol 500 mg tablet	67.5	Fairly high	88.7	High
26. Paracetamol 250 mg/5 ml syrup	60.5	Fairly high	59.3	Fairly high
27. Paracetamol 125 mg suppository	74.5	Fairly high	88	High
28. Paracetamol 100 mg tablet	64.3	Fairly high	53.7	Fairly high
Overall (range)	63.4	Fairly high	46.3	Low

##### Overall orders fill rates

**Fig. 2:**
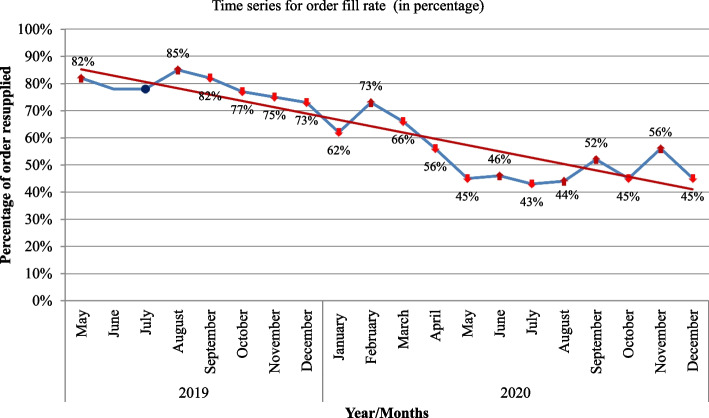
The average monthly order fill rates of hospitals

The average monthly orders fill rates of hospitals for the selected products range from 43 to 85%. Before COVID-19 the average order fill rates fulfilled the recommended fill rates of greater or equal to 70% of total orders placed by the hospitals. In August 2019, the highest (85%) order fill rate was recorded. However, starting from the month of COVID-19 case notification, the percentage of selected medicines order(s) filled correctly in terms of items and quantities started decreasing. In March 2020, when the first COVID-19 case was reported in Ethiopia, there was a significant decrease in the order fill rate of the products at health facilities. In March 2020 only 66% of orders requested were resupplied. As compared to pre-COVID-19 data, the resupply of selected items was decreased nearly by half between May 2020 and August 2020. There was a sharp decrease in stock supply starting from February 2020 through May 2020. Relatively distinct peaks followed by dramatic and even larger sharper declines in the order fill rate of drugs were evident (Fig. 2).

#### Analysis of stock out the status

##### Paracetamol product stock out the status

Table 4 shows paracetamol products' stock out status between May 01, 2019 to December 31, 2020. At least one of the four different dosage forms of selected paracetamol products listed in the Ethiopian essential medicine list (EML) was out of stock for defined periods ranging from 2 to 480 days over twenty months. The overall mean ± SD monthly stock out of paracetamol products was 7.50 ± 3.16 days. There was a statistically significant difference in monthly mean (SD) days of stock out for paracetamol 500 mg (tablet) before COVID-19 and during COVID-19. Its average monthly days of stock out were lower during COVID-19 (6.50 ± 5.82 days) as compared to the pre-COVID-19 report (11.50 ± 4.01 days) (*p* = 0.02) (Table 4).

**Table 4:** Mean monthly stock out in days for paracetamol products

Paracetamol products	Average monthly days of stockout (Mean ± SD days)			*P*-value*
	Overall	Before COVID-19	During COVID-19	
Paracetamol 500 mg tablet	7.98 ± 1.60	11.50 ± 4.01	6.50 ± 5.82	0.02
Paracetamol 250 mg/5 ml syrup	12.14 ± 4.08	11.22 ± 4.99	12.75 ± 3.45	0.12
Paracetamol 125 mg suppository	3.96 ± 3.38	5.46 ± 4.98	2.95 ± 1.11	0.52
Paracetamol 100 mg tablet	9.6 ± 9.24	1.8 ± 3.33	14.7 ± 8.22	0.04
Overall	7.50 ± 3.16	9.10 ± 4.07	6.50 ± 1.96	0.98

^*^Two tail t-test

##### Paracetamol product stock out per facilities

**Fig. 3:**
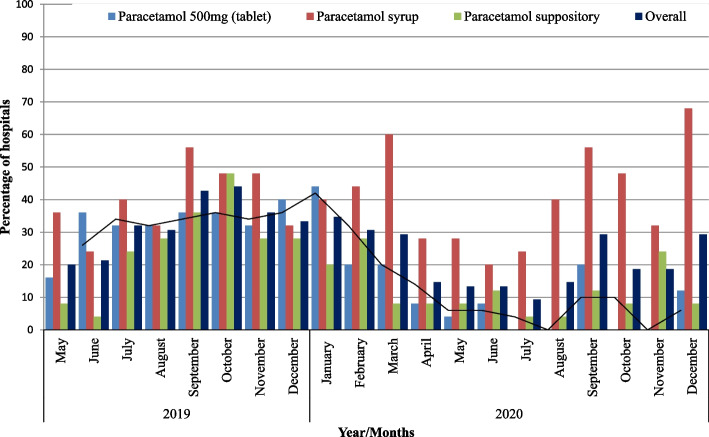
Percentage of hospitals that reported stockout of paracetamol products before and during COVID-19

The stock out period for three paracetamol products was evaluated. Relatively, the number of facilities reporting stock out for paracetamol tablets and suppository decreased starting January 2020. However, for paracetamol syrup, most of the hospitals reported significant stock out. During the pandemic, over one-third of the public hospitals experienced stock out of this product. Five months before the global outbreak of COVID-19, about one-third of the hospitals reported stock out of paracetamol adult tablets. In January 2020, 11/25(44%) of hospitals reported stock out of the product. However, the subsequent months of the pandemic, the number of hospitals experiencing stock out for paracetamol 500 mg tablet decreased substantially. Between July 2020 and November 2020, all of the hospitals had stocks of paracetamol 500 mg tablet (Fig. 3).

**Fig. 4:**
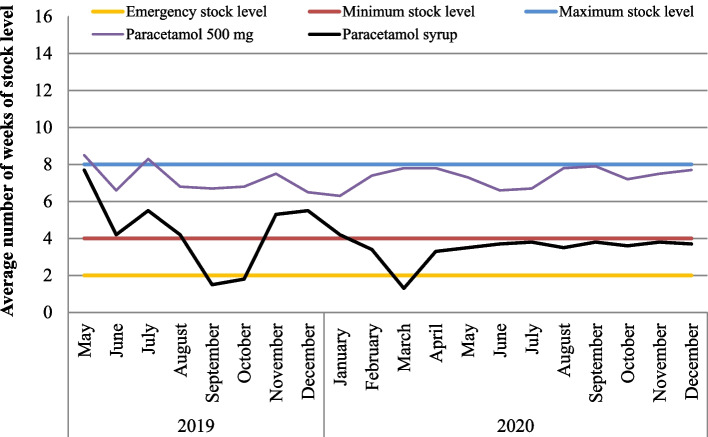
Average weeks of drug stock levels for two paracetamol products in comparison to maximum, minimum, and emergency-stock level thresholds in the hospitals

The stock status for paracetamol 500 mg (tablet) was above the 4-week minimum stock level in all months. In addition, most of the hospitals had adequate stocks of this product during the COVID-19 pandemic too. However, the stock status for paracetamol syrup was below the four-week minimum stock level starting from December 2019 and throughout all months of the year 2020, even falling below the emergency stock level threshold for three months (Fig. 4).

##### CV medicines and antidiabetics stock out the status

The hospitals experienced stock out of at least one of the three insulin products for an average of 13(9.5–18) days per month during the outbreak of COVID-19. The mean stock out period for all antidiabetics and CVD medicines was found to be greater during COVID-19 as compared to pre-COVID-19 time. From the antihypertensive products, the health facilities reported a long duration of days out of stock for methyldopa 16 (10.6–21.4) days per month (Table 5).

**Table 5:** Mean (range) monthly stock out in days for CVD medicines and antidiabetics

**Medicines products**	Average monthly day of stock out (days)		
	Overall	Before COVID-19	During COVID-19
Insulin products	10.5(7–12.5)	7.6 (4.4–13.2)	13(9.5–18)
Oral hypoglycemic agents	8.7(5- 11.5)	8.7 (2.3–12)	10.6(4–14)
Aspirin	12.5(8.2–16.8)	9 (6–14)	16(10–23)
Diuretics	6.6(4.2–9)	5.5 (3–8)	7.4 (4.2–10.6)
Beta-blockers	7.7 (6.3–9)	6.5 (5.6–7.4)	8.6 (7.8–9.5)
ACE inhibitors/ARBs	7.7(6–9.5)	5.3(2.2–8.4)	9(5–13.4)
Statins	11.6 (9.4–13.8)	8.6(6.8–10.4)	13.6(11.6–15.8)
Calcium Channel blockers	6.6 (5.3–7.9)	4.7(3.5–6.7)	7.9 (6–9.7)
Digoxin products	9.5 (5.8–13.2)	8.8 (5.6–12.4)	10.4(7.2–13.6)
Methyldopa	16 (10.6–21.4)	13(8.7–17.3)	16(14.6–21.4)

*ACE* Angiotensin-converting enzyme, *ARBs* angiotensin receptor blockers

### The impact of COVID-19 on the unit cost of selected medicines

At service delivery points, the average unit cost of medicine was determined. As a result, the range of changes was compared before and during the COVID-19 pandemic. Except for metoprolol and parenteral frusemide, the unit cost for each selected medicine showed an increment. The unit cost for all antidiabetics was increased during the COVID-19 pandemic. For example, the unit cost of metformin increased by 35.55%. Similarly, the unit cost for paracetamol products increased (Table 6).

**Table 6:** The mean (SD) changes in the unit cost (Ethiopian Birr) of selected basket medicines from government suppliers at hospitals before and during the COVID-19 pandemic

S.No	List of drug products	Pre-COVID-19	During COVID-19	Range of changes (%)^a^
1	Insulin regular(soluble) 100 units/ml	83.41 ± 1.74	93.50 ± 5.75	+ 12.09
2	NPH(insulin suspension) 100 units/ml	84.04 ± 2.02	103.05 ± 11.50	+ 22.62
3	Insulin Isophane (biphasic)(30 + 70) 100 units/ml	80.29 ± 3.19	94.51 ± 11.08	+ 17.71
4	Metformin 500 mg tablet	33.11 ± 0.00	44.88 ± 17.23	+ 35.55
5	Glibenclamide 5 mg tablet	17.95 ± 1.43	22.89 ± 6.29	+ 27.53
6	Aspiring 81 mg tablet	41.96 ± 0.00	77.90 ± 14.98	+ 85.65
7	Enalapril 5 mg tablet	42.20 ± 8.63	52.27 ± 3.49	+ 23.86
8	Captopril 25 mg tablet	60.93 ± 0.00	61.02 ± 0.06	+ 0.15
9	Atenolol 50 mg tablet	31.95 ± 0.04	37.19 ± 3.92	+ 16.38
10	Propranolol 40 mg tablet	61.81 ± 0.43	66.86 ± 1.86	+ 8.17
11	Metoprolol 25 mg tablet	143.32 ± 0.00	134.90 ± 56.02	-5.87
12	Frusemide 40 mg tablet	31.86 ± 1.71	48.21 ± 6.19	+ 51.30
13	Frusemide 10 mg/ml(injection)	86.30 ± 13.58	65.02 ± 20.69	-24.66
14	Hydrochlorothiazide 25 mg tablet	26.90 ± 0.00	35.29 ± 14.12	+ 31.19
15	Spironolactone 25 mg tablet	89.99 ± 28.01	95.93 ± 18.08	+ 6.60
16	Atorvastatin 20 m tablet	95.97 ± 28.11	175.21 ± 8.60	+ 82.57
17	Lovastatin 20 mg tablet	84.58 ± 0.00	95.96 ± 6.63	+ 13.46
18	Simvastatin 40 g tablet	49.79 ± 0.08	72.98 ± 14.96	+ 46.57
19	Losartan 20 mg tablet	112.25 ± 3.19	120.87 ± 1.82	+ 7.68
20	Methyldopa 250 mg tablet	41.93 ± 8.31	69.42 ± 14.46	+ 65.54
21	Amlodipine 5 mg tablet	27.67 ± 0.14	39.75 ± 20.75	+ 43.68
22	Nifedipine 20 mg tablet	25.66 ± 8.42	45.74 ± 11.20	+ 78.28
23	Digoxin 0.25 m tablet	137.35 ± 1.44	163.91 ± 33.31	+ 19.34
24	Digoxin 0.25 mg/ml in 2 ml(injection)	477.71 ± 0.00	480.70 ± 1.92	+ 0.63
25	Paracetamol 500 mg tablet	165.56 ± 0.00	166.54 ± 7.25	+ 0.59
26	Paracetamol 250 mg/5 ml syrup	10.57 ± 2.66	21.74 ± 5.60	+ 56.7
27	Paracetamol 125 mg suppository	110.40 ± 33.93	124.48 ± 14.48	+ 12.75
28	Paracetamol 100 mg tablet	98.33 ± 3.29	108.47 ± 4.69	+ 10.31

^a^Minus sign and plus sign indicate decrease or increase in the percentage

### Qualitative data

**Fig. 5:**
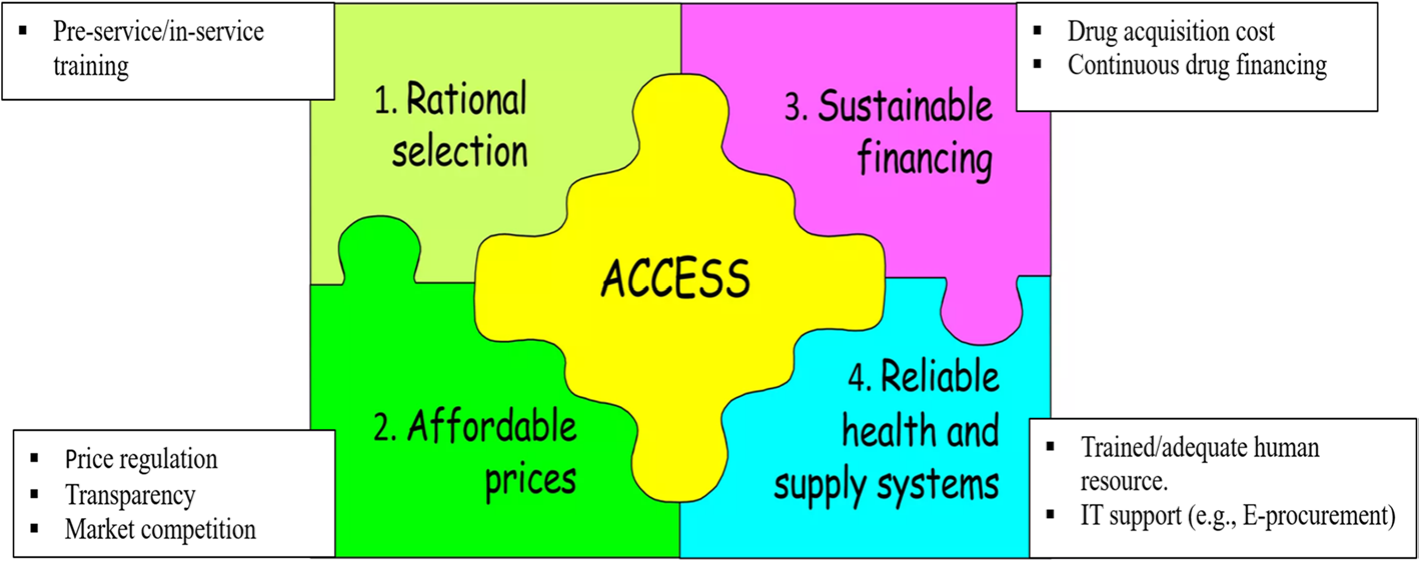
Framework for collective action for improving access to essential medicines (adapted from World Health Organization, 2004)

The qualitative assessment included fourteen (14) study sites and fifteen (15) key informants (KIs) from two stakeholders (i.e. pharmaceutical supply agencies hubs and hospitals) to explore the challenges and experiences they have been facing, as well as the interventions on the overall pharmaceutical supply systems and specifically of the related to the availability and supply of antidiabetics, CVD medicines, and paracetamol products pre-and during COVID-19 pandemic. The KI were interviewed and the results were thematically categorized into the following themes. We have considered four of the WHO frameworks for collective action for improving access to essential medicines (Fig. 5).

#### Challenges & experiences related to medicines supply

KIs were interviewed to explore the challenges they were experiencing, and interventions implemented to secure the availability and supply of antidiabetics, CVD medicines, and paracetamol products, and the results were thematically categorized into the following themes:

##### Human resource-related

The major challenge raised by most of the KIs was a human resource-related challenge. Some of the KIs from the primary hospital complained about shortages of trained human resources focused on inventory management, especially those using and documenting the LSAT were not always available at the right place to inform upon resupply decisions. One of the primary hospital’s head of pharmacy services explained the scenario as follows:

“….Before my deployment to this hospital, the hospital had no pharmacy professionals. The hospital assigned nurses to the pharmaceutical store. It was 5 months preceding the outbreak of COVID-19 when I started work. There was no appropriately documented LMIS information. I couldn't get the documents talking about the drug consumption history, requisition, and supply, such as Model-19, and Model-22 of pharmaceuticals at dispensary and store. As the time was near to the outbreak, this affected our forecasting, ordering and to make the resupply decision of the drugs during the pandemic” (27-year-old; Pharmacist from the primary hospital).

##### Drug acquisition cost and drug financing factors related

Another major challenge encountered during the pandemic was the increase in the acquisition cost of the medicines. All of the KIs from each study site complained about the increase in the unit of cost of most of the drugs and also insufficient financing allocated for drug procurement for specific facilities. The pharmacy service director of the general hospital described this challenge as:

“…The acquisition costs of drugs from the public supply agency are increasing; this has doubled during COVID-19. For example, if you take the price of NPH insulin, it was 81.6 Ethiopian Birr (ETB), before the outbreak which increased to 112.67 ETB during the pandemic. Biphasic insulin cost was increased from 68.39 ETB to 113.46. This highly affected the procurement plans we had set at the start of the budget year. Multiple times the hospital procured a limited amount of drugs (usually emergency drugs only) with the availing debt levels. If the hospital doesn’t pay the preceding debt, we cannot resupply the stock based on the need of patients. For this reason, we encountered increased emergency procurement during this COVID-19 pandemic.”(39-year-old; Pharmacist from general hospital)

##### Political instability

Some of the KIs from the remote western part of the Oromia region pointed out that political instability in the region highly affected the supply of essential medicines. There was significant disruption of telecommunications and the Internet, which are so essential for the continuity of the pharmaceutical supply and communication of LMIS tools. This challenge was explained by one of the store managers from the general hospital as follows:

"…as the telecommunication and internet were disrupted for more than three months including during COVID-19, we couldn't communicate to the supply agency for the request to resupply the medicines. This internet disruption resulted in the discontinuation of our computer software system (so-called DAGU-2), which we have been using to manage pharmaceutical products and supplies. It was not so easy to go in person to the supply agency, due to fear of security issues. When there is stock out from the public supply agency, we couldn’t find the products from the private wholesalers. This is also an impact of security issues in the region on the private sector in resupplying their stocks. This security problem compounded the underlying supply disruptions by the outbreak of COVID-19″ (33-year-old, pharmacist from general hospital).

##### Currency inflation

Pharmacists working in the Ethiopian pharmaceutical supply agency mentioned that currency inflation was also one of the major challenges affecting the continuity of pharmaceutical supply at the national level. This highly affected the supply of essential medicines to public service delivery points. One of the forecasting officers working at the branch public supply agency explained the issues as follows:

“…the value of Ethiopian birr has declined day to day; I think it is related to the political situation in the country and also international impacts of COVID-19 on the pharmaceutical system. As we are importing most of the drugs from abroad, fluctuations in the rate of dollar exchange price limited adequate supply of essential medicines. This shortage of hard currency affected not only our supply agency but also it poses difficulties on the private suppliers, who can help the public in case of central stock out”; (35-year-old, Forecasting officer from pharmaceutical supply agency)

#### Interventions for prevention of supply interruptions

##### Information technology support

Some of the general and tertiary hospital key informants mentioned the drawback of paperwork pharmaceutical services, which impacted the use of the available pharmacy workforce. The store manager of the general hospital mentioned the case as follows:

“…Bin cards and stock cards, IFRR, RRF are important documents for supply chain activity auditing, and also the indicator the pharmacy service office of Oromia regional health bureau will use to evaluate our hospital's pharmaceutical services. We usually use and update bin cards. It requires many pharmacy professionals and is time-consuming. I think, supporting pharmaceutical information management systems with information technology will ease the service provision. It is important to have a national database, which will show us the national stock and consumption status”; (37-year-old, pharmacist from general hospital).

##### Transfer from nearby health facilities

Some of the hospitals transfer some of the drugs from nearby health centers or hospitals. The nearby health facilities borrow emergency medicines from hospitals. The pharmacy service director of the general hospital (which also gives service for COVID-19 case) says:

“……………… during COVID-19 the hospital experienced stock out of different products. As the hospital focused on COVID-19 case admission and management, we don’t have medicines like metformin, insulin in the store. We transfer some stocks of these medicines from nearby hospital” (41-year-old, pharmacy service director from General hospital)

## Discussion

The World Health Assembly supported the Global Action Plan 2013–2020, which aimed for a 25% decrease in NCD-related mortality by 2025. With non-communicable diseases on the rise nationally and globally, there is an even stronger case to make for providing access to essential medicines. Among the nine global targets set to achieve this goal is the availability of affordable essential NCD medicines in both public and private facilities at a rate of 80 percent. However, data on these medicines' availability, stock out, and cost in Sub-Saharan Africa (SSA) are scarce [33]. The global pandemic further obscures and impacts the supply chain environment of these medicines. To our knowledge, this is the first study to assess the knock-on effects of the COVID-19 pandemic on the availability and stock status of selected paracetamol products, CVD medicines, and antidiabetics in Ethiopia.

The results of this study underpin the urgency to improve the availability of medicines for common chronic non-communicable diseases in the public sector, especially for inevitable outbreaks. This is vital to ensure that the prescribed CVD medicines are available and affordable. None of the surveyed chronic disease medicines met the ideal availability benchmark of 80% in the public hospitals. This was in line with multiple studies across the globe. For example, a study from Pakistan [34] showed that only 30.4% of generic essential CVD medicines were available in public sectors. Similarly, there was the availability of these medicines at public health facilities in Nigeria [35]. The current COVID-19 pandemic has exposed pharmaceutical companies’ and related actors’ supply chain weaknesses.

This study revealed that, on average, the availability of selected basket medicines was 63.4% during the pre-COVID-19 time in hospitals. During COVID-19, the availability of these medicines was 46.3% and ranges from 2.6% to 88.7%. This was relatively higher than a study done in Nigeria (35.2%) [35]. However, lower than the WHO’s Global Action Plan for NCDs targets for access to NCD medicines. This might be associated with a weak supply of essential medicines in low-income countries and also the occurrence of the COVID-19 outbreak.

More than 70–80% of Ethiopia's annual pharmaceutical consumption is imported from other countries, particularly China and India [36]. As the pandemic hit China and India, they went into lockdown, closing many factories and disrupting supply chains, affecting imports to other countries and increasing logistic constraints [37, 38]. Similarly, in early March, India imposed restrictions on exporting 13 active pharmaceutical ingredients (APIs) and 13 formulations [39], potentially further exacerbating the fragile pharmaceutical supply chain in developing countries, including Ethiopia. As much of the pharma industry in Ethiopia depends on APIs imported from these countries, Ethiopian factories have only been able to produce at limited capacities (~ 20%) of local production[36].

The COVID-19 pandemic significantly affected the availability of most CVD products. For example, methyldopa was fairly available before the outbreak. However, during COVID-19, its availability was reported to be very low. This medicine is the program drug in the maternal and child health product supplied by the donors and public supply agencies. The donor support is disrupted by the pandemic, which might affect its availability at the service delivery points.

Different countries both low and middle income as well as high income faced a shortage of paracetamol products amid rising COVID-19. Different media outlets and reports from Pakistan [40], the Philippines [41], the UK [42], USA [11, 43] showed a significant shortage of paracetamol products. Similarly, in this study, there were significant stockout reports for some of the paracetamol products specifically paracetamol syrup and paracetamol 100 mg tablet. For example, the stock status of paracetamol syrup formulations was below the minimum stock level during COVID-19 throughout the study period. Yet at the same time, paracetamol 500 mg tablets had increased availability at health facilities. This might be due to Ethiopian production. Also, the drug is used to manage COVID-19 symptoms, which might induce the facilities to include it in the emergency medicine list, thereby increasing availability. Interestingly, the price of paracetamol 500 mg was hardly changed during the pandemic, which may also point to local production stabilizing this market.

From the qualitative data from KIs, drug acquisition cost was considered as the major challenge related to the increase in stockouts and disruptions of the pharmaceutical supply, especially for the high price and out-of-pocket medicines such as CVD medicines and antidiabetics. It can be directly linked to the abrupt halt in economic activities due to lockdown and restriction of movement. The COVID-19 pandemic disrupted the national and international supply chain systems, including those of essential closures, trade restrictions among nations, and transportation problems [44, 45]. Developing countries such as Ethiopia have complicated drug supply chains with a consequent increase in drug cost as it gets to the end-user. The existence of a pandemic further compounded this problem. Developing countries acutely felt the impact of the COVID-19 pandemic on drug security as they rely heavily on imported drugs to meet their medication needs [46]. This highlights the need for policymakers in Ethiopia to address challenges to large-scale and sustainable drug manufacturing, using the COVID-19 situation as a learning opportunity.

The procurement, availability, and accessibility of pharmaceuticals is an ongoing challenge for many health systems [47, 48]. Even though multiple and complex factors influence the availability of drugs, inadequate budgetary provision, and inefficient procurement and supply systems are consistent challenges. Reports from Tanzania [49], Kenya [50, 51], Uganda [50], Ghana [50], and Mozambique [52] show budgetary and funding constraints as a key factor behind continuous medicine stockouts. This was further exacerbated by the pandemic. Allocated budgets were shifted to COVID-19 related services, resulting in the disruption of NCD management services. This might be due to the disruption of the global supply network due to COVID-19 and the focus of international partners only on COVID- 19 at the expense of other major diseases. An interim survey report from WHO, which included Ethiopia as one study site, showed that about 43 countries worldwide reported disruptions in NCD clinical services management. One of the primary reasons was the unavailability of medicines for those medical conditions [53].

COVID-19 caused a massive shock to the global economy, including commodity prices [54]. In the present study, the unit cost of most medicines increased during the pandemic. For example, the unit cost of insulin products from the government supply agency increased from 12 to 22%. Similarly, the average increase in supplier unit cost for oral hypoglycemic agents reached 35%. This was similar to the study report from Nigeria [35] Rwanda [55], and the USA [56], where the price of medicines increased during the pandemic and affected the availability and accessibility of those drugs. This might be associated with the numerous price drivers which impacted the producers. This could include operating expenses, alternative sourcing air freight charges, price rises for active pharmaceutical ingredients, and currency fluctuations [57, 58]. For example, in a recent assessment, it was estimated that the cost of antiretroviral drugs may increase by 25 percent without immediate action, which may increase the annual prices of those drugs [59].

Medicines and medical supplies are often in short supply in areas suffering from political instability. In the current study, key informants mentioned that the facilities encountered supply chain disruptions from the scourge of conflict in the western part of the country. Similarly, conflict and political instability affected the supply of essential medicines in the Middle East, like in Yemen [60]. This is linked to the poor engagement of government for the basic human needs and diversion of financial and human resources for immediate conflict-related actions and responses.

## Limitation of the study

The present study has several strengths. To the best of the authors' knowledge, this is one of the first studies assessed the supply situation and availability trends of selected generic medicines in Ethiopia prior to and during the pandemic. It employ the mixed methods approach, which will support the quantitative data about shortages of products and explored the lived experience of stakeholders, reason for shortages and potential solutions. However, there may be some possible limitations in this study. The results may not apply to all medications because the study concentrated on the availability and supply of paracetamol, antidiabetic, and CVD medications. Moreover, the study included only facilities found in seven zonal administrations, which will not reveal the situations in other settings.

## Conclusion

The COVID-19 pandemic significantly affected the supply of medicines used to treat CVD and diabetes. None of the surveyed chronic disease basket medicines met the ideal availability benchmark of 80% in the public hospitals. The overall stock out situation in the study area has worsened during COVID-19 compared to pre-COVID-19 time. However, surprisingly, the availability of paracetamol 500 mg tablets improved during COVID-19. There were multiple challenges, which compounded the impact of the pandemic on the reliable supply of affordable medicine in the study setting. These include political instability, shortage of trained human resources, currency inflation, and limited drug financing. Therefore, a range of policy frameworks and options targeting inevitable outbreaks and beyond should be strengthened to enable governments to ensure that medicines for chronic diseases are consistently available and affordable.

## Data Availability

The datasets used and/or analysed during the current study are available from the corresponding author on reasonable request.

## Abbreviations

ACEIs: Angiotensin Converting Enzyme Inhibitors
ARB: Angiotensin Receptor Blockers
APIs: Active Pharmaceutical Ingredients
COVID-19: Corona Virus Disease -2019
CVD: Cardiovascular Disease
EML: Essential Medicine List
EPSA: Ethiopian Pharmaceutical Supply Agency
ETB: Ethiopian Birr
IMAT: Inventory Management Assessment Tool
IRB: Institutional Review Board
KIs: Key Informants
LIAT: Logistic Indicators Assessment Tool
LMICs: Low and Middle-Income Countries
LMIS: Logistics Management Information System
LSAT: Logistics System Assessment Tool
NCD: Non Communicable Disease
SPSS: Statistical Package Software for Social Science
SSA: Sub-Saharan Africa
WHO: World Health Organization
